# Sirtuin 3 deficiency aggravates angiotensin II‐induced hypertensive cardiac injury by the impairment of lymphangiogenesis

**DOI:** 10.1111/jcmm.16661

**Published:** 2021-06-27

**Authors:** Chen Zhang, Na Li, Mengying Suo, Chunmei Zhang, Jing Liu, Lingxin Liu, Yan Qi, Xuehui Zheng, Lin Xie, Yang Hu, Peili Bu

**Affiliations:** ^1^ The Key Laboratory of Cardiovascular Remodeling and Function Research Chinese Ministry of Education Chinese National Health Commission and Chinese Academy of Medical Sciences The State and Shandong Province Joint Key Laboratory of Translational Cardiovascular Medicine Department of Cardiology Cheeloo College of Medicine Qilu Hospital Shandong University Jinan China

**Keywords:** cardiac remodelling, hypertension, lymphangiogenesis, Sirtuin 3, VEGFR3

## Abstract

Lymphangiogenesis is possibly capable of attenuating hypertension‐induced cardiac injury. Sirtuin 3 (SIRT3) is an effective mitochondrial deacetylase that has the potential to modulate this process; however, its role in hypertension‐induced cardiac lymphangiogenesis to date has not been investigated. Our experiments were performed on 8‐week‐old wild‐type (WT), SIRT3 knockout (SIRT3‐KO) and SIRT3 overexpression (SIRT3‐LV) mice infused with angiotensin II (Ang II) (1000 ng/kg per minute) or saline for 28 days. After Ang II infusion, SIRT3‐KO mice developed a more severe cardiac remodelling, less lymphatic capillaries and lower expression of lymphatic marker when compared to wild‐type mice. In comparison, SIRT3‐LV restored lymphangiogenesis and attenuated cardiac injury. Furthermore, lymphatic endothelial cells (LECs) exposed to Ang II in vitro exhibited decreased migration and proliferation. Silencing SIRT3 induced functional decrease in LECs, while SIRT3 overexpression LECs facilitated. Moreover, SIRT3 may up‐regulate lymphangiogenesis by affecting vascular endothelial growth factor receptor 3 (VEGFR3) and ERK pathway. These findings suggest that SIRT3 could promote lymphangiogenesis and attenuate hypertensive cardiac injury.

## 1 INTRODUCTION

Hypertensive heart injury is a constellation of abnormalities that includes systolic and diastolic dysfunction, ventricular hypertrophy and cardiac fibrosis.^1^, ^2^ Recent discoveries found out the association between cardiac injury and impaired lymphangiogenesis. Lymphatic endothelial markers were reduced in human failing heart, and lymphatic reserve was reduced in patients with heart failure with preserved ejection fraction.^3^, ^4^, ^5^ In patients with coronary artery disease, vascular endothelial growth factor C/D (VEGFC/D) was verified to be predictors of mortality.^6^ Therapeutic VEGFCc156s treatment improved cardiac lymphatic vascular function and alleviated Ang II‐induced cardiac fibrosis and dysfunction.^5^ Lymphangiogenesis also attenuated cardiac fibrosis and dysfunction after myocardial infarction.^7^ Regulating myocardial extracellular volume and alleviation inflammation underlies the protective effect of lymphangiogenesis in Ang II‐induced cardiac injury.^8^, ^9^ The most well‐studied mechanism that mediated lymphangiogenesis is the VEGFC/D‐VEGFR3 axis.^10^, ^11^ In LECs, proliferation and migration are mainly mediated by VEGFR3 and pathways such as ERK and AKT.^12^, ^13^ Based on these findings, the lymphangiogenesis may play a non‐neglectful role in modulating hypertensive cardiac injury, but the process of lymphangiogenesis in regulating Ang II‐induced cardiac injury and relative molecular mechanisms beyond cellular level has not been clarified.

Sirtuins (SIRTs) are a series of proteins belonging to the family of nicotinamide adenine dinucleotide (NAD^+^)‐dependent deacetylases. There are seven members (SIRT1‐SIRT7) concluded in sirtuins family, participating process of metabolism, oxidative stress and dynamics of mitochondria.^14^ Emerging evidence indicated that SIRT3 was an essential regulator of hypertensive cardiac dysfunction, hypertrophy and fibrosis.^15^, ^16^ Our previous study has found that SIRT3 protected heart from hypertensive heart injury by regulating metabolism and autophagy of cardiomyocyte and transdifferentiation of fibroblast.^17^, ^18^, ^19^, ^20^ Whether SIRT3 participates in the regulation of lymphangiogenesis and its effect on Ang II‐induced cardiac remodelling, however, remains unknown. It was reported that SIRT3 involved in the regulation of endothelial dysfunction and affecting the development of hypertensive cardiovascular diseases.^21^ SIRT3 also participates in proliferation and migration of endothelial cells on physiology and pathology conditions. SIRT3 expression activates human microvascular endothelial cells AKT/FOXO3a pathway activation after ischaemia‐reperfusion injury.^22^ Therefore, SIRT3 has the potential to regulate the LECs activity and promote lymphangiogenesis.

In the present study, we speculated that promoting lymphangiogenesis is another possible channel of SIRT3 to attenuate hypertensive cardiac injury. Focussing on the topic, the present study examined Ang II‐induced heart injury and lymphangiogenesis level using SIRT3 knockout and SIRT3 overexpression mice and, meanwhile, examined endothelial cell function and lymphangiogenetic capacity in primary mice LECs stimulated with Ang II. We demonstrated that SIRT3 is a positive regulator of lymphangiogenesis in Ang II‐induced hypertensive heart injury. Further, we indicated that SIRT3 promotes proliferation and migration of LECs through VEGFR3 and ERK pathways.

## 2 MATERIALS AND METHODS

### 2.1 Materials

Sirtuin 3 overexpressing lentivirus was purchased from GenePharma (GenePharma). Mini‐osmotic pump was purchased from Alzet (Durect). Ang II was purchased from Sigma (Sigma Aldrich). Mouse VEGFC ELISA kit was purchased from Meimian (Meimian). Lipofectamine 2000 was purchased from Invitrogen (Thermo Fisher). DMEM was purchased from Sciencell (Sciencell). Foetal bovine serum was purchased from Gibco (Thermo Fisher). EDU Apollo Imaging Kit was purchased from Ribobio (Ribobio). Antibodies against SIRT3, tubulin and GAPDH were purchased from Cell Signaling Technology (D22A3, 5335S and 5174S, CST). Antibody against ERK, p‐ERK and MYH6 was purchased from Proteintech (16443‐1‐AP, 80031‐1‐AP and 22281‐1‐AP, ProteinTech). Antibodies against LYVE1, VEGFR3 and α‐SMA were purchased from Abcam (ab19147, ab27278 and ab5694, Abcam). Mouse monoclonal antibody against SIRT3 was purchased from Santa Cruz (sc‐365175).

### 2.2 Animal model

All animal studies were approved by the appropriate ethics committee and performed in accordance with the ethical standards specified in the 1964 Declaration of Helsinki and its later amendments. All experiments were approved by the ethics boards of Qilu Hospital of Shandong University. Wild‐type (WT) 129 mice and SIRT3 knockout (SIRT3‐KO) mice were purchased from the Jackson Laboratory. SIRT3 overexpression (SIRT3‐LV) mice were generated by caudal vein injection of lentivirus at MOI multiplicity of infection (MOI) = 10 for 7 days, and control group mice were generated by injection of negative control lentivirus (NC‐LV). Eight‐week‐old male mice were used in the study and were housed in well‐ventilated cages under 12‐hour light‐dark cycle and fed with laboratory standard chow and water during the experiment. Mice were randomized, and ten mice were used in each group. All experimental mice anaesthetized with 1% sodium pentobarbital were implanted subcutaneously corresponding osmotic pumps beforehand filled with Ang II or saline for 28 days. The mini‐osmotic pumps were infused with chosen doses of Ang II in sterile saline (1000 ng/kg per minute) or saline. According to the directions, mini‐osmotic pumps were placed in sterile 0.9% saline at 37°C for 24 hours to prime. In brief, the experimental mice were randomly divided into 8 groups: WT + saline group, WT + Ang II group, SIRT3‐KO + saline group, SIRT3‐KO + Ang II group, NC‐LV + saline group, NC‐LV + Ang II group, SIRT3‐LV + saline group, SIRT3‐LV + Ang II group. After 28 days, mice were sacrificed at the end of the experiment and the hearts and serum were used to assay.

### 2.3 Echocardiography

Echocardiography was performed with a Visual Sonics Vevo 770 machine using a 30 MHz high‐frequency transducer (Visual Sonics). All mice were anaesthetized using 2.0% isoflurane. The left ventricular ejection fraction (LVEF), fractional shortening (FS), peak E, peak A, early (E′), late (A′), left ventricular end‐diastolic diameter (LVEDD), left ventricular posterior wall diameter (LVPWD) and electrocardiogram were measured. The ratio of early‐to‐late mitral flow velocity (E/A) and diastolic velocity ratio (E′/A′) was calculated.

### 2.4 Blood pressure measurement

Systolic blood pressure (SBP), mean blood pressure (MBP), diastolic blood pressure (DBP) and heart rate (HR) were measured by a non‐invasive tail‐cuff system (Softron). Blood pressure and HR measurements were repeated 3 times for each mouse, and mean value of each parameter was used.

### 2.5 Gravimetry

The atria and great vessels of hearts were removed to evaluate the wet weights of the ventricles. The cardiac dry weight was obtained after desiccation of the ventricles for 5 days at 65°C. The myocardial water content was calculated by the following equation: heart water content = (wet heart weight−dry heart weight)/wet heart weight × 100%.

### 2.6 Histopathology and immunostaining

The heart tissues were fixed with 4% paraformaldehyde in phosphate‐buffered saline at room temperature for 48 hours and embedded in paraffin. The heart tissues were cut into 5 μm sections for the following analyses. The sections were stained with haematoxylin and eosin (HE), and stained with wheat germ agglutinin (WGA) to measure cardiomyocyte size. Masson's trichrome staining and Sirius red staining were used to measure interstitial and perivascular fibrosis. The cardiomyocyte cell area and fibrotic level were quantified by ImageJ. The slides were incubated overnight at 4°C with the specific primary antibodies against LYVE1 (dilution, 1:200) and SIRT3 (dilution, 1:50) for immunohistochemistry and immunofluorescence. The next day, the slides were incubated with secondary antibodies for 30 minutes at 37°C to detect the expression of LYVE1 and SIRT3.

### 2.7 Cell culture

Mouse lymphatic endothelial cells (LECs) were purchased from Procell Company (CP‐M023) and grown in DMEM basic medium supplemented with 5% foetal bovine serum (FBS) and 1% penicillin‐streptomycin under an atmosphere of 5% CO_2_ at 37°C. Using Ang II treatment at appropriate dose (10^‐6^ mol/L) in culture medium for 48 hours, assay of LECs function and gene expression profile was performed. To inhibit expression of SIRT3 in vitro, the LECs were transfected with 50 nmol/L of small interfering RNA of SIRT3 (si‐SIRT3) with Lipofectamine 2000 for 48hours. The sequence of si‐SIRT3 was (5′‐3′) GGAUGUAGCUGAGCUGCUUTTAAGCAGCUCAGCUACAUCCTT. To overexpression SIRT3 in vitro, lentivirus was infected according to given manufacturer's protocol. In brief, the LECs were performed in 10 groups: control, Ang II, si‐NC + saline, si‐NC + Ang II, si‐SIRT3 + saline, si‐SIRT3 + Ang II, lv‐NC + saline, lv‐NC + Ang II, lv‐SIRT3 + saline, lv‐SIRT3 + Ang II.

### 2.8 Wound healing assay

The LECs were cultured in 6‐well plates and formed a monolayer. The 1mm‐wide linear scratch was performed in the monolayer LECs surface using yellow pipette tip and then replace culture medium with serum‐free medium with or without Ang II for 48 hours. The relative healing distance was measured in five randomly selected fields and analysed using Image J.

### 2.9 Transwell assay

The LECs were suspended in culture medium without serum at a density of 10^4^‐10^5^ cells per 200 μL culture medium and added in the upper transwell chambers. Chambers were placed into 24‐well plate which was added 800 μL culture medium with all components. After incubation for 12 hours in a humidified atmosphere containing 5% CO_2_ at 37°C, the migrated cells that had stuck to the lower surface of the membrane were fixed in 4% paraformaldehyde and stained with 0.1% crystal violet for 5 minutes. The number of migrated cells was counted in five randomly selected fields and analysed using Image J.

### 2.10 EDU staining

Lymphatic endothelial cells were seeded into 96‐well plates at a density of 10^3^‐10^4^ cells per well. Cells were incubated in medium containing EDU for 2 hours and fixed with 4% polyformaldehyde. EDU‐positive cells were labelled by Apollo and nucleus were marked with Hoechst33342. The EDU‐positive cells were visualized under a fluorescence microscope (Olympus).

### 2.11 PCR

Total RNA was extracted from fresh heart tissues using TRIzol reagent (Invitrogen) according to the manufacturer's protocols. First‐strand cDNA was produced from 1 mg of total RNA from each sample using PrimeScript RT Reagent Kit with gDNA Eraser (Takara, RR047A) according to the manufacturer's instruction. The PCR primer sequences were as follows: LYVE1, forward 5′‐TTGCCACAACTCATCCGACA‐3′, reverse 5′‐TCTGTTGCGGGTGTTTGAGT‐3′; VEGFR3, forward 5′‐ CCTGCCATACGCCACATCAT‐3′, reverse 5′‐ TGCATGAAGCCATCCTCCTC‐3′; GAPDH, forward 5′‐AGGTCGGTGTGAACGGATTTG‐3′, reverse 5′‐TGTAGACCATGTAGTTGAGGTCA‐3′. The mRNA levels of genes were analysed using TB Green Premix Ex Taq (Takara, RR420A) according to the manufacturer's instruction.

### 2.12 Western blot analysis

Appropriate protein samples from mouse ventricular tissue and LECs were run on SDS‐PAGE gel and immunoblotted with the primary antibodies, including SIRT3, MYH6, VEGFR3, LYVE1, ERK, p‐ERK, tubulin and GAPDH overnight at 4°C. The intensity of bands was measured by using ImageJ. All experiments were repeated at least three times and mean values were derived.

### 2.13 Data and statistical analysis

All statistical analyses were conducted using GraphPad Prism 8.0.2. Operator and subsequent data analysis of all experiments were blinded. The data are expressed as the mean ± standard error of mean (SEM), with centre values defined as means. Results were expressed as the mean ± SEM. Analysis of variance (ANOVA) with subsequent Scheffe's test was used to determine the significance of differences in multiple comparisons. *P* < .05 was considered statistically significant.

## 3 RESULTS

### 3.1 SIRT3 deficiency aggravated cardiac dysfunction in Ang II‐induced hypertensive cardiac injury

**FIGURE 1:**
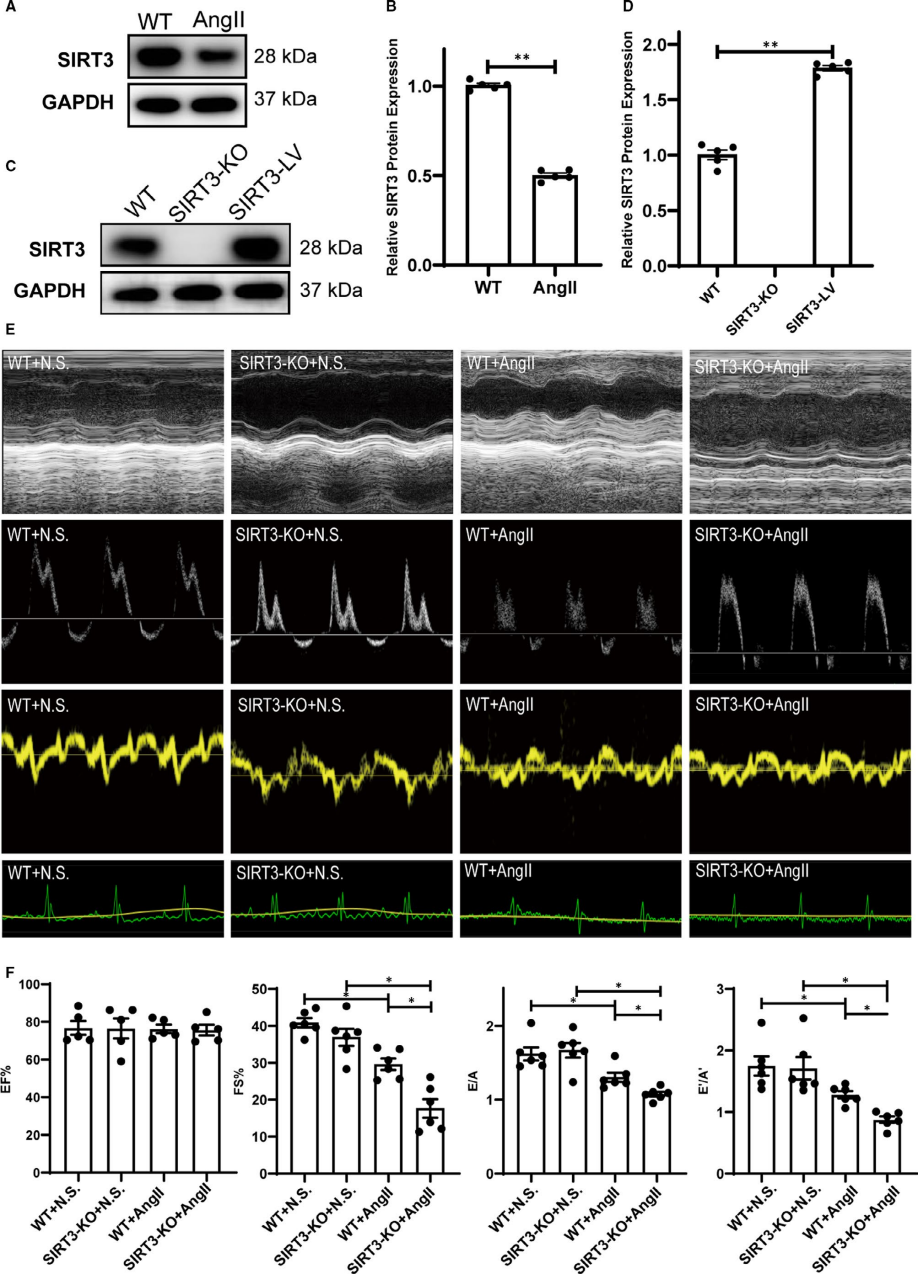
Sirtuin 3 (SIRT3) deficiency aggravated cardiac dysfunction in Ang II‐induced hypertensive cardiac dysfunction. A, Western blots of SIRT3 in saline‐ or Ang II‐treated WT mice heart. B, Statistical analysis of SIRT3 protein expression (n = 5). C, Western blots of SIRT3 in WT, SIRT3‐KO and SIRT3‐LV mice heart. D, Statistical analysis of SIRT3 protein expression (n = 5). E, Representative M‐mode, mitral inflow pulse‐wave Doppler, tissue Doppler echocardiograms and electrocardiogram of saline‐ or Ang II‐treated WT and SIRT3‐KO mice. F, Statistical analysis of EF%, FS%, E/A and E’/A’ (n = 6). (The results are expressed as the means ± SEM. The one‐way ANOVA test was used, and n represents the number of independent experiments, *P < .05, **P < .01, and ***P < .001)

To examine the effects of SIRT3 in Ang II‐induced hypertensive cardiac injury, SIRT3‐KO and WT mice were all infused with Ang II or saline for 28 days. As demonstrated, Ang II induced a significant increase in systolic blood pressure, mean blood pressure and diastolic blood pressure in WT and SIRT3‐KO mice, but the difference between SIRT3‐KO and WT was insignificant, and there were no significant differences of HR between the four groups (Figure S1). After 28 days Ang II infusion, we confirmed SIRT3 expression in mice hearts and found that SIRT3 decreased in mice exposed to Ang II infusion compared with the controls infused with saline (Figure 1A‐D). Echocardiography showed that FS%, the E/A ratio and the E′/A′ ratio in Ang II infusion group were significantly decreased than saline group and that of SIRT3‐KO mice was lower than WT mice, and the EF% did not differ between groups, indicating that SIRT3 may be involved in the mechanisms mediating hypertensive cardiac injury, especially the diastolic function damage (Figure 1E,F).

### 3.2 SIRT3 deficiency aggravated Ang II‐induced cardiac hypertrophy

**FIGURE 2:**
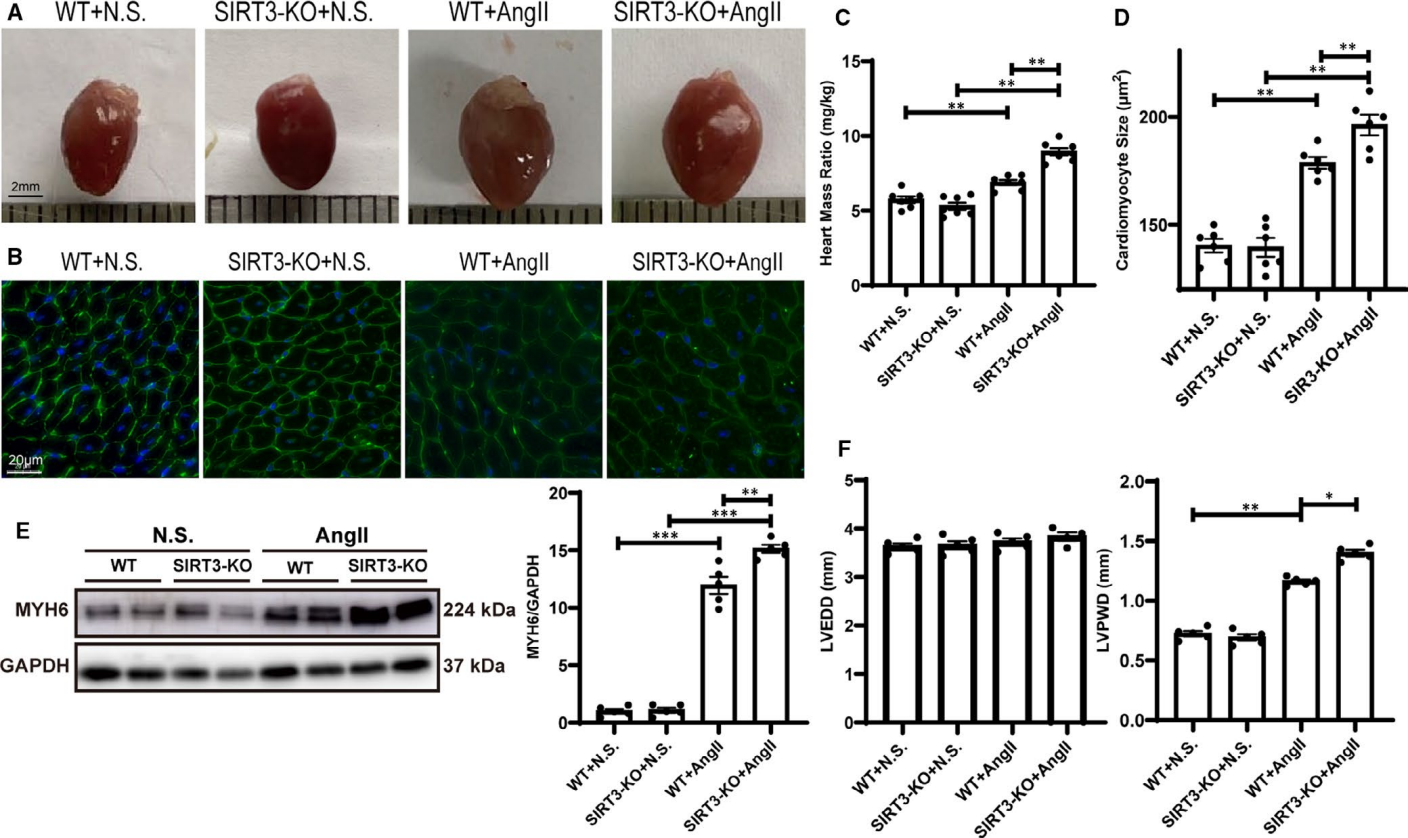
Sirtuin 3 (SIRT3) deficiency aggravated Ang II‐induced cardiac hypertrophy (A) Representative size of saline‐ or Ang II‐treated WT and SIRT3‐KO mice heart. Scale bar = 2 mm. B, Representative cardiomyocyte size by WGA staining of saline‐ or Ang II‐treated WT and SIRT3‐KO mice. Scale bar = 50μm. C, Statistical analysis of heart mass ratio, the ratio of heart weight (mg) to body weight (g) (n = 7). D, Statistical analysis of cardiomyocyte size (n = 6). (E) Western blots of MYH6 in saline‐ or Ang II‐treated WT and SIRT3‐KO mice heart and statistical analysis (n = 6). F, Statistical analysis of LVEDD and LVPWD (n = 6). (The results are expressed as the means ± SEM. The one‐way ANOVA test was used, and n represents the number of independent experiments, *P < .05, **P < .01, and ***P < .001)

To explore the effect of SIRT3 on Ang II‐induced cardiac hypertrophy, we conducted a series of experiments. We found that Ang II treatment significantly induced larger heart size than saline group, and SIRT3‐KO aggravated this change (Figure 2A). The heart mass ratio (heart weight / body weight) was increased in hypertension mice than controls, and after Ang II infusion, the heart mass ratio of SIRT3‐KO mice was higher than that of WT mice (Figure 2C). Echocardiography showed that LVPWD in Ang II infusion group was significantly increased than saline group and that of SIRT3‐KO mice was higher than WT mice, but the LVEDD showed no significant change (Figure 2F). WGA staining showed that Ang II treatment increased cardiomyocyte cell size in WT and SIRT3‐KO mice, with much significant change in SIRT3‐KO group (Figure 2B,D). Western blot showed that MYH6 protein levels were elevated in hypertension mice compared in control mice, and SIRT3‐KO up‐regulated the expression of MYH6 compared with WT group (Figure 2E).

### 3.3 SIRT3 deficiency accelerates Ang II‐induced cardiac fibrosis

**FIGURE 3:**
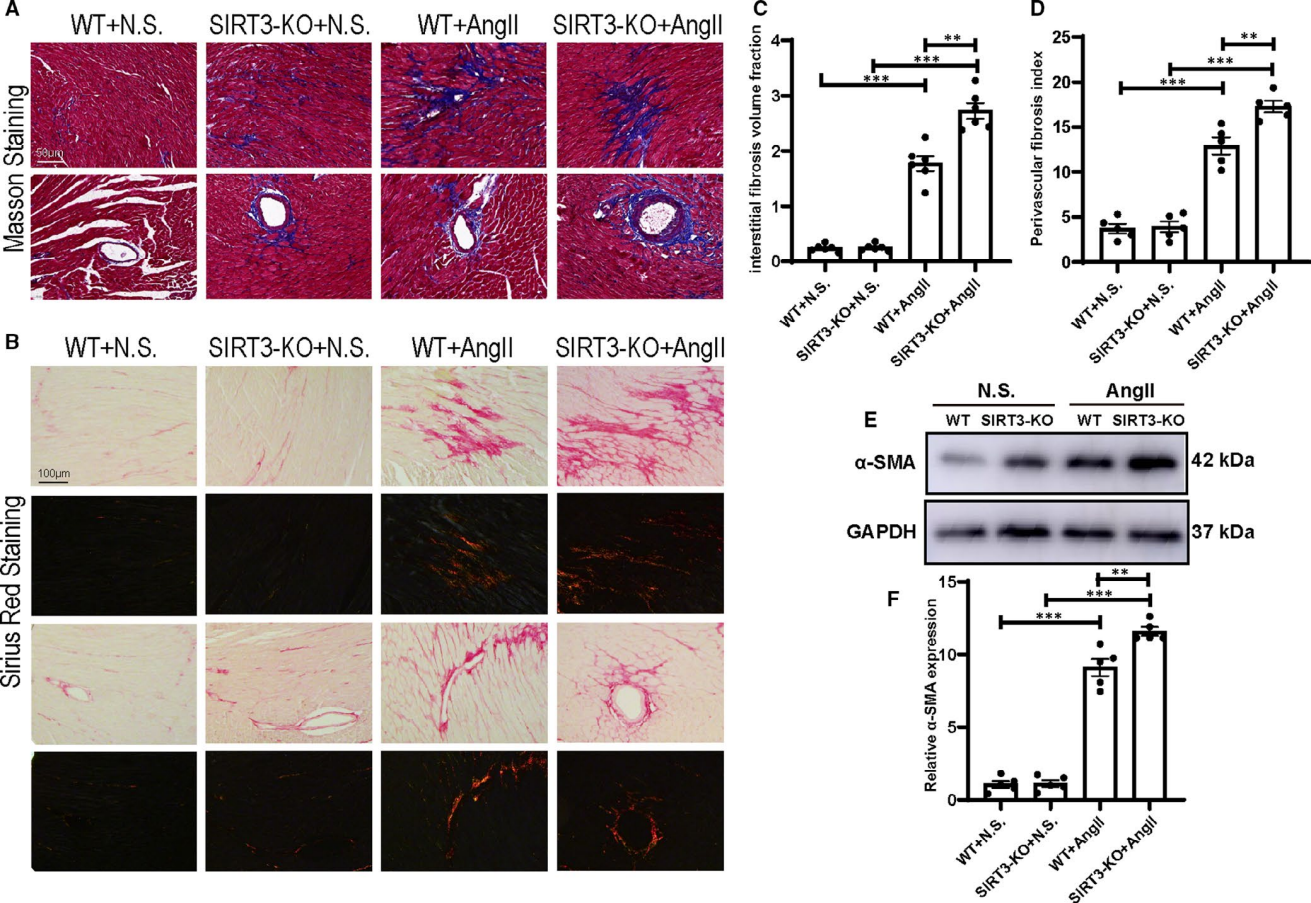
Sirtuin 3 (SIRT3) deficiency accelerated Ang II‐induced cardiac fibrosis. A, Representative images of Masson trichrome staining of saline‐ or Ang II‐treated WT and SIRT3‐KO mice heart. Scale bar = 50μm. B, Representative images of Sirius red staining of saline‐ or Ang II‐treated WT and SIRT3‐KO mice heart. Scale bar = 100 μm. C, Statistical analysis of interstitial fibrosis volume fraction (n = 5). (D) Statistical analysis of perivascular fibrosis index (n = 5). (E) Western blots of α‐SMA of saline‐ or Ang II‐treated WT and SIRT3‐KO mice heart. F, Statistical analysis of α‐SMA protein expression (n = 5). (The results are expressed as the means ± SEM. The one‐way ANOVA test was used, and n represents the number of independent experiments, *P < .05, **P < .01, and ***P < .001)

To assess the effect of SIRT3 on Ang II‐induced cardiac fibrosis, Ang II‐ or saline‐treated WT and SIRT3‐KO mice heart sections were examined with Masson trichrome staining (Figure 3A) and Sirius red staining (Figure 3B). Ang II infusion increased interstitial fibrosis volume and perivascular fibrosis index than saline group, with a higher increase in heart of SIRT3‐KO mice (Figure 3C,D). Western blot showed that α‐SMA protein levels were elevated in heart of hypertension mice compared with control, with further up‐regulated expression of α‐SMA in SIRT3‐KO mice (Figure 3E,F).

### 3.4 SIRT3 deficiency decreased cardiac lymphangiogenesis after Ang II treatment

**FIGURE 4:**
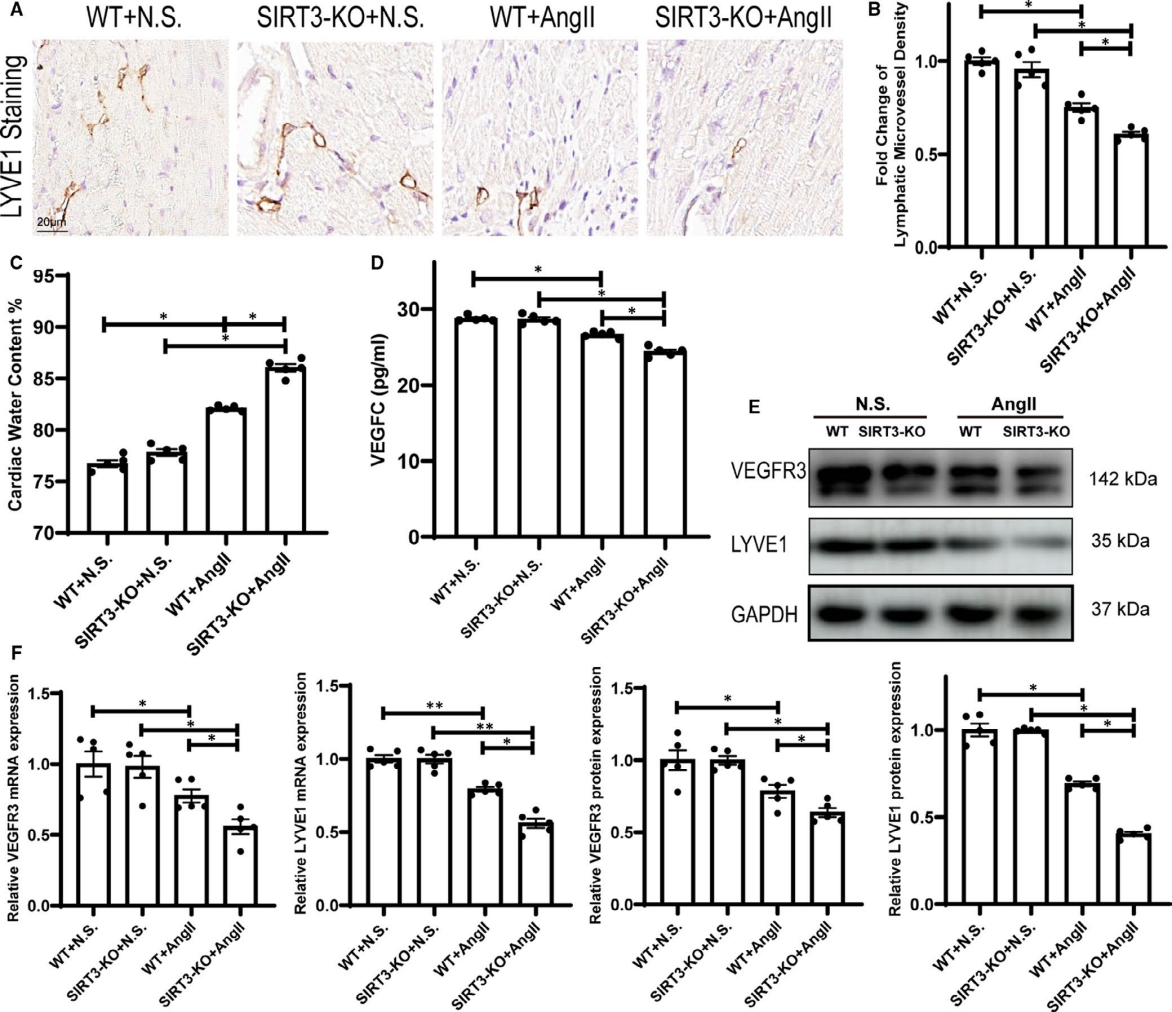
Sirtuin 3 (SIRT3) deficiency decreased cardiac lymphangiogenesis after Ang II treatment (A) Representative LYVE1 of saline‐ or Ang II‐treated WT and SIRT3‐KO mice heart. B, Statistical analysis of lymphatic microvessel density (n = 5). C, Gravimetric measurement of cardiac water content (%) (n = 5). (D) ELISA of VEGFC of saline‐ or Ang II‐treated WT and SIRT3‐KO mice serum and statistical analysis (n = 5). E, Western blots of VEGFR3 and LYVE1 of saline‐ or Ang II‐treated WT and SIRT3‐KO mice heart. F, Statistical analysis of VEGFR3 and LYVE1 mRNA and protein expression (n = 5). (The results are expressed as the means ± SEM. The one‐way ANOVA test was used, and n represents the number of independent experiments, *P < .05, **P < .01, and ***P < .001)

To verify the effect of SIRT3‐KO on lymphangiogenesis and its change in Ang II‐induced hypertensive cardiac injury, LYVE1 staining was used to mark lymphatic microvessel of heart (Figure 4A). After Ang II infusion, lymphatic microvessel density decreased compared with control group, and SIRT3‐KO mice showed lower lymphatic microvessel density than WT mice after Ang II treatment (Figure 4B). We found that Ang II treatment significantly induced cardiac oedema than saline group, and SIRT3‐KO aggravated this change (Figure 4C). ELISA assay demonstrated that mice serum VEGFC level was reduced after Ang II infusion, and SIRT3‐KO further down‐regulated VEGFC level after Ang II treatment compared with WT mice (Figure 4D). Western blot and RT‐PCR demonstrated that the mRNA and protein level of VEGFR3 and LYVE1 were down‐regulated in heart of hypertension mice compared with saline group, and SIRT3‐KO further down‐regulated the mRNA and protein expression in hypertension model (Figure 4E,F).

### 3.5 SIRT3 regulated function of LECs

**FIGURE 5:**
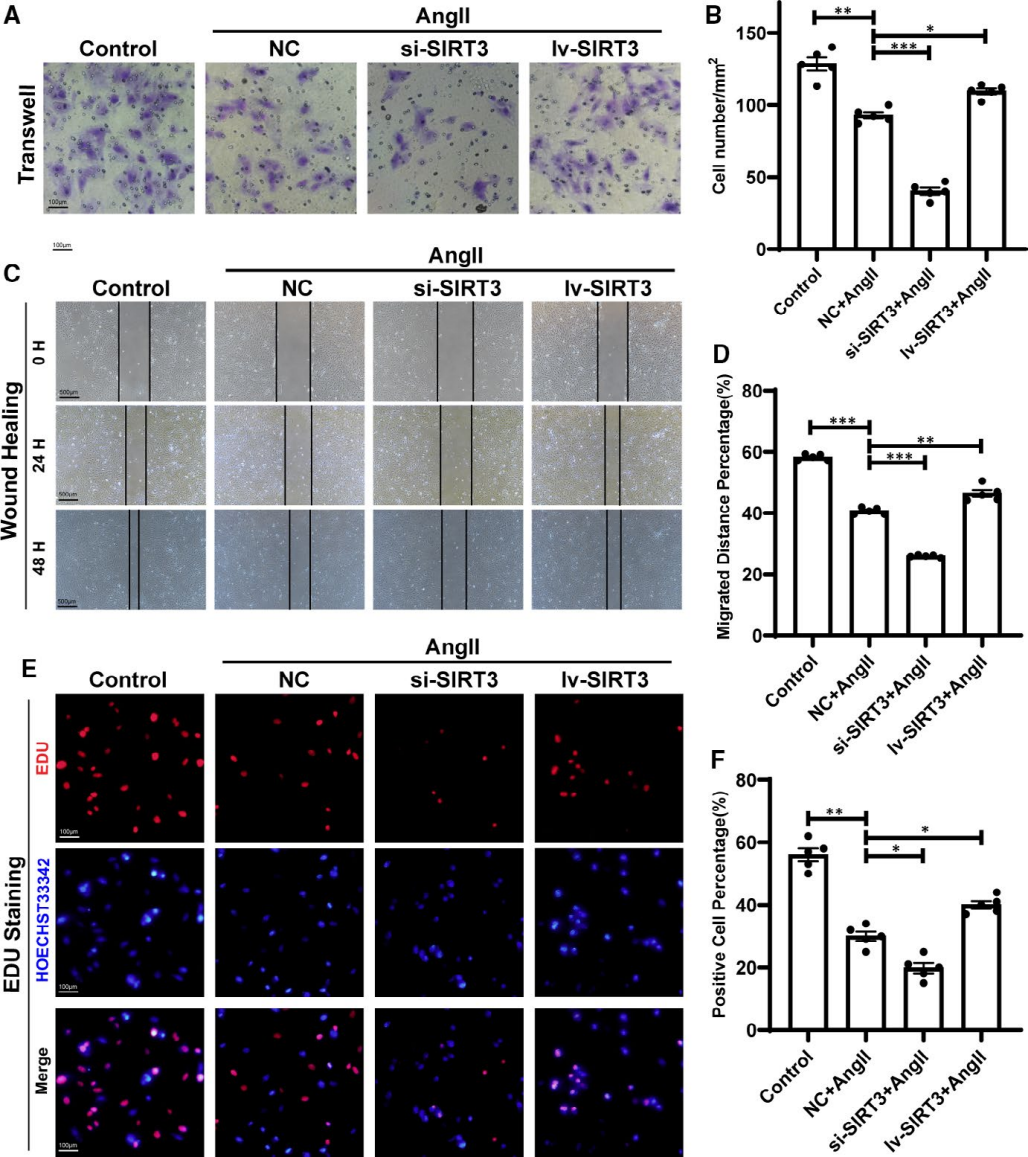
Sirtuin 3 (SIRT3) regulated migration and proliferation of LECs. A, LECs migration was detected by transwell assay. Scale bar = 100 μm. B, Statistical analysis of migrated cells number (n = 5). C, LECs migration was detected by wound healing assay. Scale bar = 500 μm. D, Statistical analysis of relatively healing distance (n = 5). E, LECs proliferation was detected by EDU staining. Scale bar = 100 μm. F, Statistical analysis of EDU staining (n = 5). (The results are expressed as the means ± SEM. The one‐way ANOVA test was used, and n represents the number of independent experiments, *P < .05, **P < .01, and ***P < .001)

To further explore the effect of SIRT3 on lymphatic endothelial function of Ang II‐treated LECs in vitro, we performed transwell assay and wound healing assay to examine migration capacity. As instructed, migrated cell number in transwell assay and healing area were all down‐regulated after Ang II exposure compared with controls. After Ang II treatment, small interfering RNA of SIRT3 (si‐SIRT3) transfection decreased the migration of LECs; conversely, lentivirus of SIRT3 overexpression (lv‐SIRT3) infection increased this process (Figure 5A‐D). EDU staining assay was performed to examine proliferation. As shown in Figure 5E,F, EDU‐positive rate was decreased after Ang II treatment, and the si‐SIRT3 transfection further decreased the proliferation of LECs, while lv‐SIRT3 increased the proliferation (Figure 5E,F).

### 3.6 SIRT3 promote lymphatic marker expression in LECs and regulated ERK pathway

**FIGURE 6:**
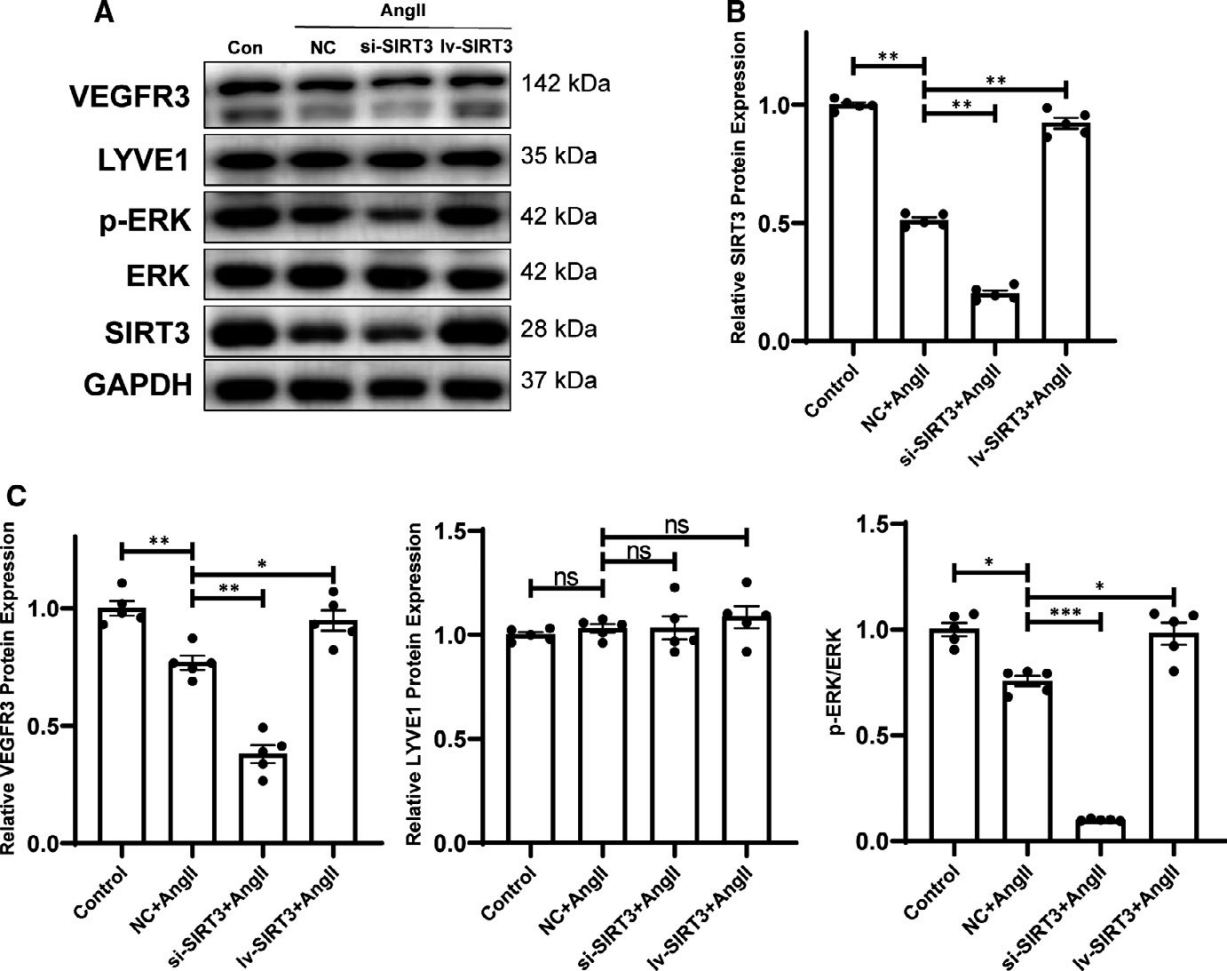
Sirtuins 3 (SIRT3) promoted lymphatic marker expression in LECs and regulated ERK pathway. A, Western blots of VEGFR3, LYVE1, p‐ERK, ERK, SIRT3 in mice LECs and statistical analysis (n = 5) (B) Statistical analysis of SIRT3 protein expression (n = 5). C, Statistical analysis of VEGFR3 protein expression, LYVE1 protein expression and ERK phosphorylation (n = 5). (The results are expressed as the means ± SEM. The one‐way ANOVA test was used, and n represents the number of independent experiments, *P < .05, **P < .01, and ***P < .001)

To confirm the molecular mechanism participated in the effects of SIRT3 on LECs, protein levels of lymphatic markers such as VEGFR3 and LYVE1 expressed in LECs were examined by Western blot. VEGFR3 and LYVE1 protein expression were reduced after Ang II administration. After Ang II exposure, inhibition of SIRT3 decreased VEGFR3 protein expression level, while overexpression of SIRT3 reversed. However, the change of LYVE1 after SIRT3 inhibition or overexpression was not significant (Figure 6A‐C). Western blot indicated that the ratio of p‐ERK to ERK was down‐regulated after Ang II treatment. Inhibition of SIRT3 decreased p‐ERK/ERK ratio, while overexpression of SIRT3 reversed. The results indicated that the effect of SIRT3 on lymphangiogenesis include VEGFC‐VEGFR3 axis and ERK pathway (Figure 6A‐C).

### 3.7 SIRT3 overexpression up‐regulated cardiac lymphangiogenesis and reversed hypertensive cardiac remodelling

**FIGURE 7:**
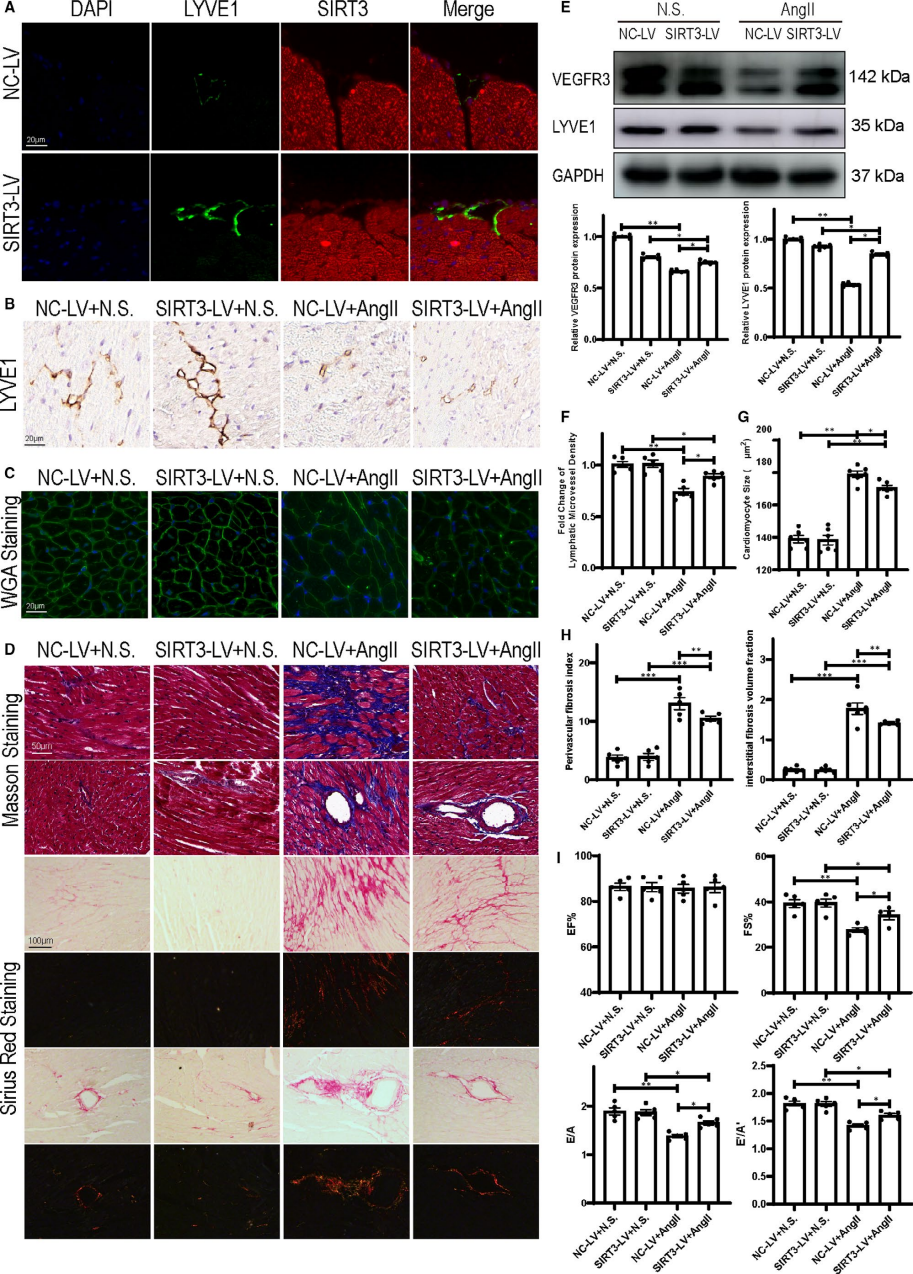
Sirtuin 3 (SIRT3) overexpression up‐regulated cardiac lymphangiogenesis and reversed hypertensive cardiac remodelling (A) Immunofluorescence staining represents LYVE1 and SIRT3 expression of NC‐LV and SIRT3‐LV mice heart. B, LYVE1 staining represents lymphatic microvessel density of saline‐ or Ang II‐treated NC‐LV and SIRT3‐LV mice heart. C, WGA staining represents cardiomyocyte size of saline‐ or Ang II‐treated NC‐LV and SIRT3‐LV mice heart. Scale bar = 50 μm. D, Representative images of Masson trichrome staining of saline‐ or Ang II‐treated NC‐LV and SIRT3‐LV mice heart. Scale bar = 50μm. Representative images of Sirius red staining of saline‐ or Ang II‐treated NC‐LV and SIRT3‐LV mice heart. Scale bar = 100 μm. E, Western blots of VEGFR3 and LYVE1 in mice heart and statistical analysis (n = 5). (F) Statistical analysis of lymphatic microvessel density (n = 5). G, Statistical analysis of cardiomyocyte size (n = 6). (H) Statistical analysis of interstitial fibrosis volume fraction and perivascular fibrosis index (n = 5). (I) Statistical analysis of EF%, FS%, E/A and E’/A’ (n = 5). (The results are expressed as the means ± SEM. The one‐way ANOVA test was used, and n represents the number of independent experiments, *P < .05, **P < .01, and ***P < .001)

To test whether SIRT3‐induced lymphangiogenesis attenuated hypertensive cardiac, SIRT3 overexpression mice and NC mice were infused with Ang II for 28 days. Immunofluorescence staining showed SIRT3 overexpression in cardiac lymphatic vessels (Figure 7A). As LYVE1 staining demonstrated, SIRT3 overexpression increased the lymphatic microvessel density and the expression of lymphangiogenesis marker of heart. (Figure 7B,E,F). WGA staining showed that SIRT3 overexpression reversed Ang II‐induced cardiomyocyte hypertrophy (Figure 7C,G). Masson trichrome staining and Sirius red staining indicated that SIRT3 overexpression reversed Ang II‐induced interstitial and perivascular fibrosis (Figure 7D,H). Echocardiography of LVEF, FS, the E/A ratio and the E′/A′ ratio examination also indicated that cardiac diastolic function of SIRT3 overexpression mice was preserved (Figure 7I).

## 4 DISCUSSION

Hypertension has been recognized as a significant health problem worldwide, and hypertensive cardiac injury is one of its most important complications. The microcirculation, especially lymphatic microvessel circulation, plays an important role in protecting the heart from cardiac injury.

A variety of cellular and molecular signalling mechanisms are involved in the pathogenesis of hypertensive cardiac injury, in particular the renin‐angiotensin‐aldosterone system (RAAS). Previous studies have found that Ang II cause hypertension and hypertensive cardiac hypertrophy and fibrosis.^23^, ^24^ SIRT3 can protect the heart from multiple types of diseases which cause cardiac hypertrophy and fibrosis, through the regulation of oxidative stress, metabolism and aging.^14^, ^25^, ^26^, ^27^ It was consistent with the previous study that SIRT3 deficiency aggravated Ang II‐induced heart injury. SIRT3 knockout mice exhibited a more severe cardiac hypertrophy and fibrosis after Ang II infusion, and these mice also exhibited increased cardiomyocyte size with an upregulation of MYH6 expression, increased interstitial and perivascular fibrosis, and upregulation of α‐SMA expression. However, SIRT3 overexpression attenuated cardiac injury. These results strongly suggest that SIRT3 is involved in the development of cardiac injury after Ang II infusion.

Recent studies have found that the lymphangiocrine system is essential for cardiac growth, repair and cardioprotection.^28^ Using LYVE1 and VEGFR3 as markers for LECs, our data further indicated that the abnormalities caused by SIRT3‐KO may result from a decreased density of lymphatic microvessels. Although there has been no research looking at the effect of SIRT3 on lymphangiogenesis, the origin of the LECs tends to be similar to venous endothelial cells.^29^ SIRT3 protects the heart from endothelial dysfunction through the regulation of oxidative stress and metabolic reprogramming.^30^ Therefore, we suspect that SIRT3 has a similar role in the protection of LECs. Lymphangiogenesis is regulated by the VEGFC‐VEGFR3 axis and the ERK pathway.^31^ In the present study, we found that Ang II‐induced hypertension inhibited lymphangiogenesis and down‐regulate the expression of lymphatic markers in mice hearts and decrease VEGFC in their serum, and SIRT3‐KO further suppressed these processes. In vitro, Ang II reduced function, expression of VEGFR3 and phosphorylation of ERK in LECs, which could be further down‐regulated by inhibition of SIRT3, but up‐regulated after SIRT3 overexpression, suggesting VEGFC‐VEGFR3 axis, and the ERK pathway may be associated with SIRT3‐regulated lymphangiogenesis.

Sirtuin 3 represents a widely distributed mitochondrial deacetylase in various cells and can regulate glycolipid metabolism. The energy metabolism of the lymphatic vessels mainly depends on fatty acid oxidation, and during the process of intravenous endothelial to lymphatic endothelial transition, a large amount of acetyl coenzyme A is necessary.^32^, ^33^ Therefore, behind this phenomenon, there may be a more profound mechanism involving SIRT3 regulation of lymphangiogenesis. Furthermore, we observed a positive effect of SIRT3 on VEGFC‐VEGFR3, where this growth factor is found includes myocardial cells, fibroblasts and macrophages, but the specific mechanism of action of SIRT3 upregulating VEGFC has yet to be elucidated. In addition, it was reported that low‐dose, short‐time administration of Ang II induced compensatory lymphangiogenesis in heart.^34^ However, lymphangiogenic function was decreased in decompensatory stage of hypertensive cardiac remodelling with inflammation, fibrosis and heart failure.^5^ Our study validated the latter. Lymphatic vessel and hypertensive heart disease is an area that has been less studied at present. In our study, we found that micro‐lymphatic vessel density was negatively correlated with cardiac oedema, hypertrophy and fibrosis in the hypertension‐induced heart injury mice model, which may be through the influence of inflammatory response, lymphatic drainage and metabolic production.^11^

Our study firstly explored the relationship between SIRT3 and lymphangiogenesis, and its function in hypertensive cardiac injury. SIRT3 and lymphangiogenesis may represent a new therapeutic target for the attenuation of hypertension and cardiac remodelling. It has been found that an increasing number of chemotherapeutic drugs lead to heart disease, such as tyrosine kinase inhibitors (TKIs), which often inhibit angiogenesis and lymphangiogenesis, causing hypertension and cardiac dysfunction.^35^, ^36^, ^37^ Furthermore, previous studies found a series of activator of SIRT3, such as resveratrol and melatonin, ameliorate endothelial dysfunction.^38^, ^39^ Therefore, the activation of SIRT3 and lymphangiogenesis may represent a new treatment idea to reduce the incidence of heart disease caused by chemotherapy drugs.

**FIGURE 8:**
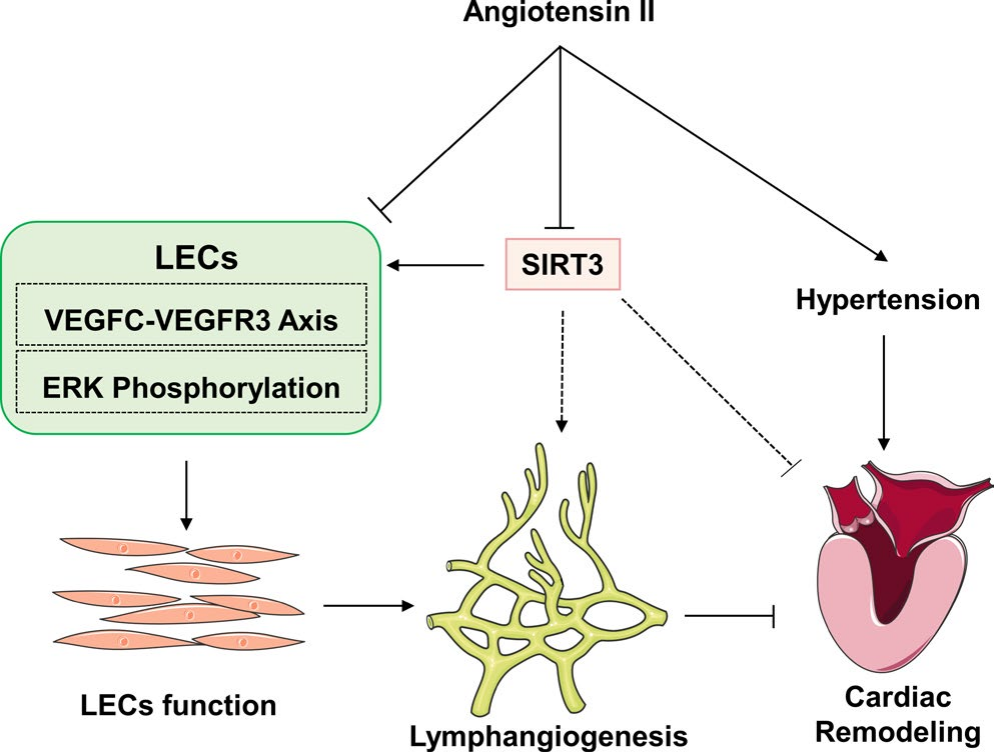
Proposed model for the role of Sirtuin 3 in hypertensive heart remodelling. SIRT3 enhanced VEGFC/VEGFR3 axis and phosphorylation of ERK in LECs, thus promoted LECs function and lymphangiogenesis. Ang II‐induced hypertension, impaired cardiac lymphangiogenesis and caused cardiac remodelling. SIRT3 protects heart from hypertensive cardiac injury by promoting lymphangiogenesis

In summary, SIRT3 protect heart from Ang II‐induced cardiac injury by promoting lymphangiogenesis, by upregulating the VEGFC‐VEGFR3 axis and increasing migration and proliferation in LECs (Figure 8).

## CONFLICT OF INTEREST

The authors declare that there is no conflict of interest.

## AUTHOR CONTRIBUTIONS

**Chen Zhang:** Conceptualization (lead); Investigation (lead); Methodology (lead); Writing‐original draft (lead). **Na Li:** Conceptualization (supporting); Software (lead). **Mengying Suo:** Data curation (supporting); Investigation (supporting); Writing‐review & editing (equal). **Chunmei Zhang:** Software (supporting); Validation (lead). **Jing Liu:** Methodology (supporting); Visualization (lead). **Lingxin Liu:** Data curation (lead); Validation (supporting). **Yan Qi:** Formal analysis (supporting); Resources (lead). **Xuehui Zheng:** Formal analysis (lead); Visualization (supporting). **Lin Xie:** Data curation (supporting); Writing‐review & editing (equal). **Yang Hu:** Data curation (supporting); Writing‐review & editing (equal). **Peili Bu:** Funding acquisition (lead); Project administration (lead); Supervision (lead).

## Supporting information

Fig S1

## Data Availability

All data generated or analysed during this study are included in this article.
