# Supplementary figures and images for: Sirtuin 3 deficiency aggravates angiotensin II‐induced hypertensive cardiac injury by the impairment of lymphangiogenesis
Source: J Cell Mol Med. 2021 Jun 27;25(16):7760–71. doi: 10.1111/jcmm.16661 (PMC8358873; doi:10.1111/jcmm.16661)

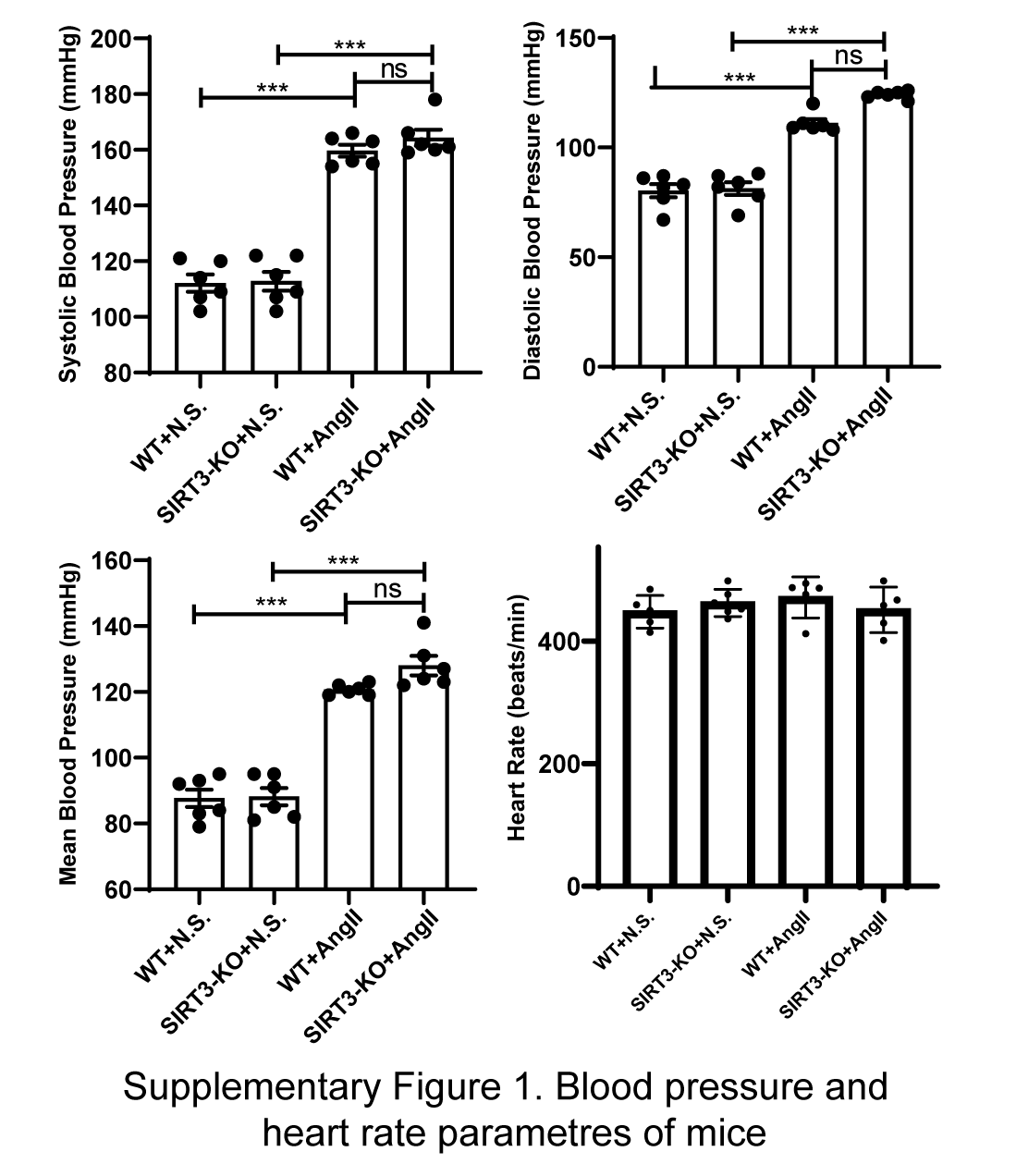

Supplement: Supplementary file 1 — Fig S1 [file JCMM-25-7760-s001.tif]
